# Robotic-Assisted Rehabilitation and Spinal Neuromodulation After Spinal Cord Injury: From Mechanisms to Trial-Informed Practice

**DOI:** 10.3390/jcm15093401

**Published:** 2026-04-29

**Authors:** Valerio Pisani, Emanuela Covella, Sergio Di Fonzo, Valeria Di Pasquale, Caterina Garcovich, Emanuela Lena, Marta Mascanzoni, Giorgio Scivoletto

**Affiliations:** 1 Spinal Center, IRCCS Fondazione Santa Lucia, 00179 Rome, Italy; e.covella@hsantalucia.it (E.C.); s.difonzo@hsantalucia.it (S.D.F.); v.dipasquale@hsantalucia.it (V.D.P.); c.garcovich@hsantalucia.it (C.G.); e.lena@hsantalucia.it (E.L.); 2 Spinal Rehabilitation (SpiRe) Lab, IRCCS Fondazione Santa Lucia, 00179 Rome, Italy; 3 Department of Human Sciences, LUMSA University of Rome, 00193 Rome, Italy; m.mascanzoni@lumsa.it; 4 Spinal Unit, CTO “Andrea Alesini” Hospital, 00145 Rome, Italy; giorgio.scivoletto@aslroma2.it

**Keywords:** spinal cord injury, robotic-assisted rehabilitation, spinal neuromodulation, neuroplasticity

## Abstract

Spinal cord injury (SCI) is an acute, devastating neurologic condition that results in permanent progressive motor deficits, sensory disturbances, and autonomic dysfunctions, which limit function, participation, and quality of life. Although substantial progress has been made during the last several decades for both early trauma care and rehabilitation protocols following SCI, long-term neurological recovery remains unpredictable and often incomplete. This manuscript summarizes mechanistic and clinical evidence regarding robotic-assisted rehabilitation (RAR) and spinal neuromodulation (SN), which have been published since 2010 until the present time in a structured narrative review of the literature on these two emerging areas for neurorehabilitation after SCI. RAR provides high-intensity, task-specific training that consistently results in improvements in functional outcomes such as balance, coordination, and independence; however, its impact is limited when it comes to walking speed or voluntary motor control. SN (particularly epidural stimulation) can activate the residual neural pathways to standing up and stepping even after a complete injury but effects are typically stimulus dependent, with heterogeneous clinical results that often lack strong long-term evidence due in part to variability in patient selection, stimulation parameters and rehabilitation protocols. However, there is emerging mechanistic data supporting combining modulation of excitability through SN approaches along with structured sensorimotor training as an approach for enhancing recovery. Collectively, these findings support a shift toward more physiology-driven neurorehabilitation strategies and the need for future research to improve clinical translation and outcome predictability by patient stratification using standardized intervention protocols that include longitudinal evaluation.

## 1. Introduction

Spinal cord injury (SCI) is one of the most disabling neurological conditions in rehabilitation, affecting not only movement but also autonomic system regulation, standing balance, bowel and bladder function, and ultimately participation in life. Millions of people worldwide live with chronic disability due to SCI, and the prevalence is increasing despite a small decrease in incidence [1]. Long-term management of SCI across all phases of care remains largely based on comprehensive rehabilitation strategies [2].

From a clinical perspective, the major challenge is not only survival, but the recovery of motor function that leads to independence (e.g., standing, stepping, reaching, or stabilizing the trunk). Modern neurorehabilitation has advanced from compensating for a loss of function to reactivating neural circuits, because spinal networks remain functionally latent even years after injury if appropriately stimulated [3,4].

From a mechanistic standpoint, the vision of rehabilitation that directly engages residual connectivity (i.e., preserved neural pathways that remain functionally inactive but structurally intact), drives plastic reorganization, and produces functional recovery even years after injury is now reachable with two technological paradigms: robotic-assisted rehabilitation (RAR) and spinal neuromodulation (SN) [5,6,7]. Building on foundational work on sensorimotor restoration, it has become clear that spared pathways can reorganize after injury, given appropriate training conditions [8].

Robotic exoskeletons can deliver very intense, repetitive, task-specific training and provide proprioceptive input that can impact spinal motor output [5]. In parallel, electrical stimulation techniques, particularly epidural or transcutaneous spinal stimulation, can alter the excitability of spinal circuits and produce volitional movement patterns that were previously believed to be irreversibly lost [6,7].

However, clinical outcomes remain variable, and gains in walking speed are not always correlated with ambulatory status [9]. Although there is growing evidence of recovery potential, improvements observed in controlled lab settings do not always translate into functional independence in real-world contexts.

In current practice, these technologies are frequently used in parallel rather than in an integrated manner. Robotic training provides structured sensory input, but may be insufficient when voluntary activation is limited [10]. Conversely, neuromodulation can enable motor output, but maintaining motor output stability frequently necessitates concurrent task-specific training [11].

As a result, each approach addresses only a portion of the neurophysiological mechanism underlying recovery, and rehabilitation techniques that do not integrate both aspects are unlikely to produce long-term gains. Previous studies have made an indirect attempt to bridge this gap.

Exoskeleton training improves functional metrics such as balance and motor scores, but gain in speed and endurance have not been reliably demonstrated [12]. Similarly, epidural stimulation has enabled standing and stepping in individuals with complete paralysis, although performance is highly dependent on context and stimulation parameters [13,14].

These experiments show independent stepping and improved postural responses, which are consistent with activation of residual spinal networks [6,15]. They also combine stimulation and training. Instead of defining a clinical framework, they show what is possible because they are diverse, often few in number, and mechanistically fragmented.

From a systems perspective, this variability has practical implications. Clinicians are required to choose among costly technologies with little to no clear comparative benefit because there is no clear integration strategy between rehabilitation and devices. Patients may experience improvements in controlled settings, but these gains are not sustained in real-world mobility settings.

Such outcome variability makes cost-effectiveness and rehabilitation technology adoption more difficult from a systems perspective [16]. The field is therefore characterized by a paradox: strong evidence of recovery mechanisms coexists with limited guidance for clinical implementation.

A mechanistically informed synthesis is needed to connect clinical decision-making, technology, and physiology. The literature increasingly supports the view that recovery may occur when neuromodulation establishes a permissive neural state, which robotic training can then shape into functional motor patterns [10,17].

However, a structured framework for outlining how and when these interventions should be integrated is still lacking.

Here we critically review recent advances in RAR and SN after SCI integrating mechanistic insights from preclinical studies with clinical trial evidence. We investigate neurophysiological mechanisms of recovery in response to robotic training and SN, compare clinical outcomes across the trials using each intervention alone or in combination, identify sources of variability, and propose a trial-informed conceptual framework for incorporating these approaches into rehabilitation practice. This review follows a structured narrative approach. Relevant literature was identified through database searches and prioritized according to methodological rigor and clinical relevance.

### Plain Language Summary

Spinal cord injury (SCI) often leads to severe and long-lasting disability, affecting movement, independence, and quality of life. New rehabilitation approaches aim not only to compensate for lost function but also to reactivate remaining neural pathways. We will review two emerging strategies: Robotic-assisted rehabilitation (RAR), which employs devices such as exoskeletons to deliver intensive, repetitive movement training to improve coordination, balance, and independence, but not always to the point of restoring voluntary movement, and spinal neuromodulation (SN), which employs electrical stimulation of the spinal cord to activate residual neural circuits to elicit movements, even in patients with very severe paralysis, although often only when the stimulation is ongoing. The take-home message of this review is that these two approaches may be complementary, with neuromodulation “awakening” the nervous system and robotic training “molding” movement into functional activity. The outcomes, however, are highly variable among patients, and long-term benefits remain to be determined; further research is needed to identify which patients will benefit most and how best to optimize treatment protocols.

## 2. Search Strategy and Selection Criteria

Although this manuscript was designed as a structured narrative review that integrates mechanistic and clinical evidence on RAR and SN following SCI, it was not conceived as a systematic review. Consequently, the PRISMA methodology was not applied, and therefore, a formal study selection flow diagram is not provided.

A targeted literature search was performed to identify relevant studies published between 2010 and 2026 by searching MEDLINE/PubMed, Scopus, and Web of Science using combinations of Medical Subject Headings (MeSH) and free-text terms including “spinal cord injury”, “robotic rehabilitation”, “exoskeleton”, “locomotor training”, “epidural stimulation”, “transcutaneous spinal cord stimulation”, “neuromodulation”, “brain-spine interface”, and “neuroplasticity”.

Publications were included if they were randomized controlled trials, prospective cohort studies, pilot studies, systematic reviews, meta-analyses, and selected preclinical or translational studies that provided a mechanistic interpretation of recovery, as well as case reports when they provided unique mechanistic or clinical insights, primarily in the area of epidural stimulation.

Articles not published in English and studies focused on compensatory orthotic devices that do not have a neurorehabilitation or neuroplastic rationale were excluded. As the available evidence is highly heterogeneous across populations in lesion characteristics, intervention protocols, stimulation parameters, training intensity, and outcome measures, a formal quantitative meta-analysis was not conducted. Instead, the evidence was qualitatively synthesized.

To rank each of these studies in terms of relevance for the mechanistic–clinical integration of RAR and SN, we used four predetermined criteria: (1) methodological hierarchy, with higher weight given to randomized and controlled clinical studies; (2) sample size; (3) direct relevance to the mechanistic–clinical integration of RAR and SN in a rehabilitation setting; and (4) priority to publications, with more recent evidence (when multiple studies addressed similar questions) contextual to the field’s evolution retained.

Results are presented per intervention domain (RAR, SN, and combined approaches), focusing mainly on functional outcomes, mechanistic interpretation, translational challenges, and implications in terms of trial-informed rehabilitation strategies. The level of evidence reported in the tables within text was derived using a simplified hierarchy based on study design: randomized controlled trials and meta-analyses were considered high-level evidence; cohort studies or systematic reviews were classified as moderate-level evidence, and case series, pilot studies, or single-subject reports as low-level evidence.

## 3. Critical and Analytical Literature Review

Over the years, rehabilitation for SCI has evolved from compensatory approaches to restorative treatments based on neuroplasticity (i.e., the nervous system’s ability to change in response to experience). Recent findings from modern neurophysiology show that spinal networks can still generate coordinated motor output when provided with appropriate sensory input and excitability modulation [3,4], with important clinical implications: recovery is not a spontaneous event but an activity-dependent phenomenon that specific treatments can influence.

These concepts have led to two technological paths: RAR, which offers high-intensity, repeatable, task-specific movement training with structured afferent feedback, and SN, which modulates the excitability of residual spinal circuits to enable volitional motor activity previously thought lost forever [10,17].

Although both interventions show functional promise in their own right, they may represent a mechanistic continuum from neural activation through the induction and facilitation of motor learning. In particular, the central research question for both treatments is not whether they work, but rather how these two different types of treatment interact to produce durable functional recovery. While randomized controlled trials provide moderate-level evidence for improvements in functional classification, most mechanistic interpretations derive from small-cohort or observational studies.

### 3.1. Robotic-Assisted Rehabilitation (RAR)

The primary goal of robotic exoskeletons was to deliver higher levels of locomotor training beyond the scope of a therapist. Initial clinical studies showed that these systems were both feasible and usable; for example, people with thoracic motor-complete SCI using a powered exoskeleton were able to walk continuous distances and at velocities close to the limits of regular ambulation [18]. They were able to learn device control within a few sessions [19].

Subsequent research focused on functional outcomes using more robust measures. However, many early trials lacked comparative controls and had limited sample sizes, restricting the ability to systematically evaluate safety and efficacy. Although there was a trend toward improvement in the ambulatory classification after 12 weeks of exoskeleton training [9], consistent with studies comparing functional classification with quantitative performance measures, gait speed did not change significantly.

From a mechanistic perspective, meta-analytic evidence suggests that robotic therapy leads to greater gains in balance, lower-limb strength, and functional scores than traditional therapies; however, no difference has been found for walking speed or distance, likely because of the underlying mechanism of action, which involves continuous proprioceptive input as well as repetitive activation of locomotor circuits [12].

Early theoretical models proposed that robotic training may directly influence spinal plasticity mechanisms [20]. Prospective comparative studies have reported differential functional responses between robotic-assisted and conventional rehabilitation paradigms [21].

A large inpatient randomized controlled trial found that overground robotic exoskeleton training did not show superiority over conventional gait rehabilitation in the overall cohort. However, subgroup analyses revealed greater functional gains in individuals with AIS C injuries, suggesting that responsiveness may depend on residual motor connectivity [22].

Earlier meta-analyses similarly reported improvements in functional outcomes with powered exoskeletons, although effect sizes varied considerably across studies [23]. Systematic evaluations of gait speed outcomes have also highlighted substantial variability in performance gains [24].

Recent randomized data suggest that response is highly dependent on training dose. In a randomized, controlled trial in chronic incomplete SCI comparing different frequencies of robotic-assisted gait training, greater training intensity led to significantly greater improvements in endurance, balance, and functional mobility [25].

In addition to reductions in spasticity immediately after training sessions, increased functional independence and life satisfaction have been reported. However, alterations in bowel, urinary, or long-term neurological function have been inconsistent [26]. Locomotor improvements appear to be related to baseline residual stepping ability [9], suggesting that robotic training primarily reinforce residual circuitry, while evidence for restoration of descending control remains limited.

In line with these findings, wearable overground exoskeleton training has also been associated with dose-dependent reductions in spasticity and parallel improvements in functional independence, as measured by the Spinal Cord Independence Measure. These findings reinforce the role of high-intensity repetitive gait training in modulating both motor and non-motor outcomes [27].

However, patient response remains heterogeneous. Gait speed appears to be influenced by injury characteristics and training parameters, with limited evidence that device type alone determines performance [24]. Biomechanical analyses suggest that loading conditions during gait are influenced by body weight support [28], indicating that RAR does not bypass physiological limitations such as reduced neural drive.

Beyond controlled trials, feasibility studies further clarify the role of wearable robotics in clinical practice across different stages of injury. Although it is difficult to separate spontaneous recovery from training effects, neurologically controlled exoskeleton training in acute SCI has been proven to be safe and associated with improvements in walking capacity and motor scores [29].

Individuals with paraplegia or tetraplegia have reported high acceptability for standing, transfers, and functional exercises requiring minimal therapist assistance using self-stabilizing powered devices [30]. Qualitative investigations during acute rehabilitation also indicate high patient engagement and perceived functional benefit [31].

Comparative crossover trials suggest that, when used within supervised training programs, powered exoskeletons may improve walking efficiency and reduce physiological demand during gait compared to other forms of gait aids [32].

Additional clinical studies support functional improvements in gait performance and independence in incomplete SCI, although heterogeneity in protocols and patient selection remains a limiting factor for generalizability [33]. These results are consistent with systematic reviews, demonstrating heterogeneity in outcome measures and patient selection.

Evidence from Lokomat-based interventions further supports improvements in walking function and activity levels. However, translation into community walking remains debated [34], supporting the view that robotic systems are effective for repetitive tasks rather than as independent contributors to neurological recovery [35].

Interestingly, not all robotic-assisted interventions lead to strength improvements. A pilot randomized study using a robotic device for targeted hip flexor training showed no significant gains in muscle strength compared with control conditions [36].

Narrative syntheses of RAR emphasize its potential for delivering high-dose training while acknowledging limitations in mechanistic interpretation [37]. For example, after structured training programs, individuals with chronic thoracic motor-complete spinal cord injury have demonstrated the ability to ambulate with assistance using powered exoskeletons.

The results of key clinical studies on robotic-assisted rehabilitation are summarized in Table 1, highlighting variability in patient populations, intervention protocols, and functional outcomes.

### 3.2. Spinal Neuromodulation (SN)

Research on neuromodulation has led to some of the most striking functional findings in SCI rehabilitation. For example, epidural stimulation has demonstrated that individuals with motor-complete paraplegia could regain weight-bearing standing and voluntary movement when spinal circuitry is in an optimal excitability state [6]. Lumbosacral epidural stimulation has also been shown to establish a brain–spinal interface, enabling independent standing in individuals with chronic motor-complete paralysis [38].

Still, other studies reported that stimulation combined with structured locomotor practice can facilitate independent stepping and maintain motor control [11]. Recent multimodal approaches further support this interaction. In the RESTORES trial, epidural stimulation combined with mental imagery and robotic-assisted rehabilitation enabled recovery of volitional motor control and overground walking even in individuals with chronic motor-complete SCI, suggesting that integrated paradigms may enhance the functional relevance of neuromodulation [39].

More recently, reports of spontaneous muscle activation without assistance for walking or overground ambulation within days after implantation in chronic injury indicate preserved but functionally silent connectivity below the lesion [14].

From a mechanistic perspective, stimulation does not directly generate movement but modulates the excitability of spinal sensorimotor circuits, enabling them to translate proprioceptive and residual descending pathways into coordinated output [7,15]. Stimulation alone rarely produces long-term functional carryover because it does not provide the patterned sensory input that can stabilize emerging motor activity during training [17]. This interaction is thus reciprocal: stimulation allows movement, and training organizes it into behavior.

**Table 1:** Summary of clinical studies on robotic-assisted rehabilitation in patients with spinal cord injury.

Study	Study Design	Level of Evidence	N	Population	Intervention	Duration	Outcomes	Key Findings
Edwards et al. (2022) [9]	RCT	High	25	Chronic incomplete SCI	Exoskeleton gait training	12 weeks	Walking speed, balance	Improved functional classification; no significant improvement in walking speed
Prończuk et al. (2026) [25]	RCT	Moderate-High	75	Chronic incomplete SCI	Robot-assisted training	12 weeks	Gait, balance, endurance	Improved gait, balance, and endurance
Aach et al. (2023) [29]	Cohort	Moderate	50	Acute SCI	HAL exoskeleton	12 weeks	Walking capacity, motor score	Safe and associated with improved motor outcomes
Chen et al. (2022) [32]	Multicenter trial	Moderate	40	Mixed SCI	Exoskeleton training	4–8 weeks	Gait parameters	Improved gait efficiency and reduced effort
Pournajaf et al. (2026) [27]	Clinical study	Moderate	74	SCI	Wearable powered exoskeleton	5 to 15 sessions	Spasticity	Dose-dependent reduction in spasticity
Baunsgaard et al. (2018) [26]	Observational	Low	13	Chronic SCI	Exoskeleton training	8 weeks	QoL, spasticity	Improved quality of life and reduced spasticity
Lillelund Sørensen et al. (2026) [36]	Pilot RCT	Moderate	12	Incomplete SCI	ROBERT^®^ robotic training	8 weeks	Muscle strength (hip flexors)	Improved muscle strength
Khadour et al. (2026) [33]	Clinical Study	Moderate	46	Incomplete SCI	Exoskeleton-assisted walking	5 weeks	Walking function	Improved walking ability and functional mobility
Swank et al. (2025) [22]	RCT	High	106	Subacute Incomplete SCI	Overground robotic exoskeleton training	Subacute phase	Gait performance	Improved gait outcomes during inpatient rehabilitation

Abbreviations: SCI, spinal cord injury; RCT, randomized controlled trial; QoL, quality of life; HAL, Hybrid Assistive Limb; N, number of participants.

A significant division in this area is between epidural spinal cord stimulation (EES) and transcutaneous spinal cord stimulation (tSCS). EES requires the surgical implantation of electrodes placed over the dorsal side of the spinal cord for spatial targeting and continuous modulation of neural excitability, which can produce powerful motor responses due to its proximity to the neural structures. It has led to standing, stepping, and voluntary rotation production in individuals with severe paralysis [6,11,14].

A recent systematic review confirms the expanding clinical interest in epidural stimulation while highlighting persistent heterogeneity in stimulation parameters and outcome reporting [40]. Implantation is surgically risky, with individualized parameters needed for optimization; clinical outcomes depend on residual connectivity and the training environment.

In contrast, tSCS uses surface electrodes to pass current through less region-specific overlapping spinal circuits, which can also affect reflex pathways, voluntary movement amplitude, and locomotor performance [7]. Real-world data support the feasibility and clinical applicability of transcutaneous approaches.

A recent community-based pilot study combining multisite tSCS with activity-based therapy demonstrated both safety and functional improvements, highlighting the potential scalability of non-invasive neuromodulation strategies [41]. Single-subject designs have reported improvements in locomotor and autonomic domains with multisite transcutaneous stimulation paradigms [42].

Repetitive applications can produce longer-lasting physiological adaptations; however, experimental paradigms suggest that these changes are most effective when combined with training to improve walking outcomes. The non-invasive nature of tSCS makes it far more clinically accessible and scalable than implanted systems, although its effects are generally subtler and more task-specific.

This distinction between the two modalities reflects a broader principle. While epidural stimulation provides the higher amplitude and more immediate physiological activation, often resulting in faster gains in individuals with severe paralysis, transcutaneous stimulation offers repeatable modulation over longer time scales and can be applied with greater safety.

Both approaches rely on behavioral training after therapy application, and translation into routine clinical practice remains limited and uneven across centers. Short-term clinical applications of spinal stimulation have shown meaningful improvements in functional outcomes alongside positive patient-reported experiences, emphasizing the relevance of patient-centered endpoints in neuromodulation research [43].

A recent systematic review of neuromodulation trials identified several ongoing studies but relatively few published outcome datasets [44]. Most studies rely on small cohorts with heterogeneous stimulation parameters, rehabilitation protocols, and outcome measures, limiting generalizability and cross-study comparison.

Pilot investigations in sensorimotor-complete populations continue to explore feasibility and individualized parameter optimization [45]. Recent comprehensive reviews outline both the therapeutic potential and translational challenges of neuromodulation strategies in SCI [46].

A representative example includes patients with chronic motor-complete SCI who regained the ability to stand and perform voluntary movements under epidural stimulation, demonstrating the presence of functionally latent neural pathways.

Key clinical studies on spinal neuromodulation are summarized in Table 2, illustrating differences in modality, study design, and functional outcomes.

### 3.3. Combined Approaches: Toward Mechanistic Integration

Combining robotic training with neuromodulation has also been explored. Cervical transcutaneous stimulation in combination with an upper-limb exoskeleton has been shown to increase motor strength and hand function compared with exoskeleton training alone [47]. A closed-loop neuroprosthetic system synchronized with robotic movement led to long-term neurological gains and more physiologically relevant muscle activation patterns [10]. Likewise, multimodal rehabilitation combining stimulation and locomotor training has enabled independent stepping in individuals with motor-complete SCI [11].

These findings suggest that neuromodulation may create a permissive neural state, while robotic training develops patterned motor output. When integrated, these interventions may become functionally relevant for recovery by targeting both excitability and sensorimotor learning components of motor control.

Animal studies support this interpretation. Brain–spinal interface-based gait restoration in primates [48] has shown that when cortical intention is combined with spinal stimulation, bypassing the lesion can maintain physiological movement patterns, consistent with hierarchical models of distributed motor control. Experimental rodent models previously established restoration of voluntary locomotion through targeted neuromodulation strategies [49].

The literature consistently indicates that robotic interventions tend to produce high-quality movement but rarely restore true volitional control. In contrast, neuromodulation can enable movement, often through stabilization mechanisms, but typically requires subsequent training for functional use.

Preliminary evidence suggests that combined approaches may enhance recovery compared with single-modality interventions, although controlled comparative trials remain limited. However, variability in outcomes remains substantial. Some patients regain mobility, whereas others with similar injury characteristics do not [13].

Improvements in walking may occur without a measurable increase in speed [9], and gains in functional improvements may not translate into independence [26]. These discrepancies likely reflect differences in patient selection and variability in intervention protocols.

There is significant heterogeneity in methodological quality. Many neuromodulation studies are case-based, while robotic trials often lack detailed mechanistic endpoints. In addition, the cost-effectiveness of these interventions remains inconclusive [16].

In this context, it is useful to distinguish between biological and clinical plausibility. Biological plausibility refers to an intervention’s ability to modulate neural circuits and generate motor output under controlled conditions, whereas clinical plausibility refers to the reproducibility of these effects as meaningful functional improvements in real-world settings.

For example, neuromodulation studies consistently demonstrate activation of motor output under controlled stimulation conditions. However, translation into sustained functional independence remains inconsistent across trials.

Overall, the evidence for biological plausibility is stronger than for clinical plausibility. This discrepancy highlights persistent gaps in integrating physiology into intervention design and clinical decision-making.

Research tends to evaluate technologies in isolation, whereas functional recovery depends on their interactions. The literature lacks a unified framework linking mechanisms (neural excitability) and training parameters (sensorimotor input) to clinical outcomes (functional independence).

To better contextualize variability in clinical outcomes, a comparative synthesis of functional domains across interventions is presented in Table 3.

### 3.4. Population Burden and the Need for Mechanistically Guided Rehabilitation

The scale of disability following SCI may explain the relative failure of incremental gains in rehabilitative technologies. While incidence rates for SCI are relatively stable, global epidemiological data indicate that prevalence is rising. Updated global epidemiological analyses confirm the sustained burden of SCI worldwide over the past two decades [50]. The burden of disability remains particularly high among older people with cervical-level SCI [1].

The persistence and magnitude of disability after SCI suggest that incremental improvements in compensatory care have not reduced long-term dependency. As a result, the clinical question has now shifted from managing impairment toward modulating residual neural function.

This shift aligns with the modern understanding of spinal motor organization. Locomotion is not generated solely by descending cortical commands but emerges from distributed spinal central pattern generator networks that integrate supraspinal intent with sensory feedback [4]. Classic experimental work has demonstrated that spinal circuits retain intrinsic rhythm-generating properties after injury, provided that appropriate sensory and excitability conditions are restored [51].

Experimental and clinical rehabilitation studies show that when appropriate afferent input and excitability modulations are provided, motor output can be elicited even in the most impaired patients [3]. This observation supports the rationale for technological rehabilitation strategies that seek to control physiological state rather than compensate for structural loss.

Although initial studies in robotic ambulation focused on mobility, more recent work has begun to suggest an even greater physiological effect. Exoskeleton-supported gait can activate autonomic regulatory pathways [52], improving bowel function and microbiota composition, alongside psychosocial and functional benefits during powered walking [18].

Additional randomized pilot studies suggest potential improvements in lower urinary tract function following exoskeleton-assisted gait training [53]. Improvements in pulmonary function parameters have also been reported [54], and cardiovascular adaptations during prolonged robotic locomotor programs have also been documented [55].

Robotic training of the upper limb has also been shown to benefit motor coordination. For example, wrist-robot interventions have been shown to improve movement kinematics (i.e., the quality and pattern of movement) independently from increases in strength or corticospinal excitability [56]. Similarly, combined RAR in tetraplegia has produced functional improvements comparable with conventional occupational therapy but more focused on prehension quality [57].

Further insight into these mechanisms is provided by biomechanical and modeling investigations [58], which demonstrate that the performance of powered exoskeletons depends strongly on the interaction between user biomechanics and mechanical assistance.

Machine-learning analyses of robotic gait training [28] have shown that body weight support and loading parameters are key determinants of functional symmetry outcomes. These findings suggest that robotic efficacy is less dependent on device sophistication and more on the appropriateness of physiological input conditions.

### 3.5. Neuromodulation Across Modalities: From Reflex Modulation to Brain–Spine Interfaces

The range of neuromodulation research now extends from non-invasive stimulation to brain-controlled neuromodulation systems [7]. Depending on stimulation frequency and context, experimental data demonstrate modulation of reflex gain, voluntary movement amplitude, and locomotor range [7]. These findings suggest that neuromodulation primarily affects network excitability, rather than directly movement itself.

At the invasive end of this spectrum are brain–spine interfaces, which directly link cortical activity to spinal stimulation. In primate models, such systems have enabled weight-bearing locomotion within days after injury [48]. Reviews of digital neuromodulation platforms (i.e., advanced systems integrating stimulation with computational control) have proposed that similar interface may be scaled for restoration via residual pathways [59].

The same physiological principle underlies clinical stimulation studies. Recovery appears to occur when there is synchrony between functional motor intent and neural activation.

Importantly, the efficacy of any form of neuromodulation depends on concurrent training. Experimental rehabilitation protocols combining stimulation and sensorimotor therapy have been shown to be more effective than either intervention alone [17]. This supports the interpretation that neuromodulation creates a permissive neural state. At the same time, experience determines behavior within this state.

### 3.6. Integrated Rehabilitation: Evidence for Interaction Effects

A substantial body of evidence supports the role of combined approaches in promoting functional recovery. Examples include cervical stimulation plus robotic upper-limb training [47], hybrid functional electrical stimulation–exoskeleton systems [60], and closed-loop neuroprostheses synchronized with robotic movement [10].

Across these technologies, findings are consistent with locomotor training findings showing persistent improvement years after injury when high-intensity activity-based therapy is provided [61]. These results suggest that recovery is not a function of the modality delivering task-specific feedback but rather an interaction between neural excitability and task-specific feedback.

Despite encouraging results, outcomes remain highly variable. Some patients achieve overground walking following stimulation, whereas others with a similar injury classification do not.

A systematic review suggests that the number of neuromodulation studies is increasing, although published findings remain limited and endpoints are highly heterogeneous [44]. This discrepancy is likely attributable to differences in residual connectivity rather than inconsistent treatment effects.

This interpretation is consistent with findings in hand dexterity recovery following spinal cord injury, where the paradigm has shifted from lesion completeness toward physiological stratification, emphasizing spared indirect pathways and network reorganization [62].

Economic analyses add another layer of complexity to the challenge of translating this knowledge for application in rehabilitation and research. RAR is still considered experimental [16]. Although some studies suggest potential cost-effectiveness, results remain inconsistent due to heterogeneous protocols, outcome measures, and the absence of reliable patient selection criteria.

Recent evidence has convincingly shown modifiable functional changes (i.e., neural pathways whose activity can be altered by therapy) in spinal circuits after injury. However, this research remains limited by methodological heterogeneity.

From a methodological perspective, robotic studies have stronger methods of trial and a weaker mechanism of interpretation. In contrast, neuromodulation studies offer clearer mechanistic insight but poorer generalizability to human patients.

Combined approaches, although limited in number, appear to be more aligned with the requirements for functional recovery. Accordingly, the literature is now beginning to support the idea that effective rehabilitation is not device-dependent but rather physiology-dependent.

The next challenge is to translate this mechanistic understanding into reproducible clinical protocols. This requires integrating trial outcomes with mechanistic insight, moving from experimental demonstration toward standardized therapeutic strategy.

Taken together, the available evidence suggests that functional recovery after SCI may depend less on the isolated efficacy of a given modality and more on the interaction between neural excitability and task-specific sensorimotor input.

Although robotics and neuromodulation have often been investigated independently, emerging combined paradigms indicate that modulation of spinal network excitability may enhance responsiveness to structured training. However, variability in patient selection, stimulation parameters, and outcome measures continues to limit generalizability.

**Figure 1:**
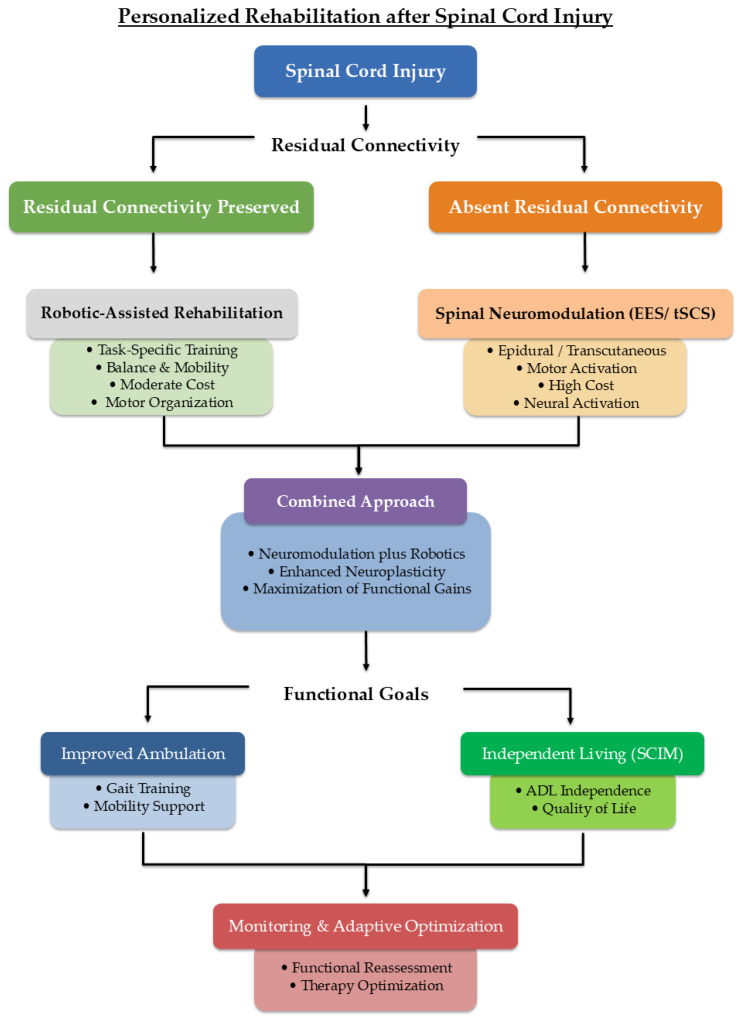
Personalized rehabilitation after spinal cord injury. The figure shows a conceptual decision-making framework based on residual connectivity. Robotic-assisted rehabilitation supports motor organization via task-specific training, while spinal neuromodulation (epidural or transcutaneous) promotes neural activation; combined approaches may enhance neuroplasticity and maximize functional recovery. Outcomes are measured using measures such as the Spinal Cord Independence Measure (SCIM), with iterative monitoring and adaptive optimization of therapy.

In light of these findings, we propose a hypothesis-generating clinical decision-making framework that integrates injury characteristics, residual connectivity, and functional status to guide the sequential or combined application of RAR and SN (Figure 1).

The framework is conceptual and is intended to support stratified trial design and clinical reasoning rather than prescriptive implementation.

## 4. Discussion

This review aimed to integrate mechanistic and clinical evidence regarding RAR and SN in SCI. To facilitate clinical interpretation, the following discussion integrates mechanistic findings with their practical implications for patient care.

For example, a patient with incomplete spinal cord injury may benefit from robotic training to improve walking stability, while neuromodulation may enhance the ability to initiate movement. Rather than evaluating these technologies as independent interventions, the available literature suggests that functional recovery may emerge from the interaction between modulation of neural excitability and structured sensorimotor training.

This perspective shifts the emphasis from device efficacy alone toward understanding how physiological state and task-specific input interact within residual spinal networks. The discussion therefore focuses on interpreting areas of convergence and divergence across studies and identifying factors that may account for variability in clinical outcomes.

Several RAR trials have established measurable functional improvement without proportional restoration of motor capacity [9,12]. Recent high-quality trials further support this interpretation. Robotic-assisted training improves functional outcomes, but only in certain subpopulations, such as AIS C patients, does it significantly outperform conventional therapy at the group level, according to extensive randomized data [22], particularly with higher training intensity.

Although early feasibility studies indicated that ambulation was safe and associated with psychosocial benefits [18,19], these gains plateaued even with additional training. Across different study designs, this pattern appears to reflect a physiological rather than methodological effect.

The most likely explanation is that exoskeletons do not generate voluntary motor drive but rather provide repetitive proprioceptive input that reinforce locomotor patterns already encoded within spinal circuitry [3]. As a result, improvements in independence measures may occur alongside relatively small changes in raw performance metrics.

In contrast, neuromodulation studies show an opposite pattern. These studies demonstrate recovery of motor activation without sustained functional integration. Epidural stimulation has enabled individuals classified as motor-complete [6,14] to stand and voluntarily move, and task-specific stimulation has allowed stepping in long-term paralyzed subjects [11].

These findings suggest the presence of functionally latent pathways that can be reactivated. However, motor output is generally maintained only with additional stimulus and training, both of which are necessary for continued functional performance.

From a systems perspective, this apparent contradiction between robotic and neuromodulatory outcomes can be reconciled. Neuromodulation enhances the excitability of spinal networks, enabling residual inputs to generate motor output, whereas robotic training structures this output-reproducible behavior.

Thus, the two approaches act at different levels of motor control, activation versus organization. Current evidence supports the hypothesis of a complementary interaction between the two approaches.

Although fewer combined interventions have been performed, studies integrating stimulation with locomotor training have shown more meaningful recovery. Examples include multimodal rehabilitation enabling independent walking in patients with complete paralysis [11], and closed-loop stimulation synchronized with robotic movement resulted in sustained improvement [10].

Collectively, these findings support a complementary interaction model in which neuromodulation may increase the excitability of spinal circuits, while robotic training may help structure and stabilize emerging motor patterns. Although direct comparative evidence remains limited, this interpretation is consistent with both experimental and early translational data.

Rather than positioning recovery as device-driven, the available literature suggests that functional outcomes may depend on the temporal and physiological alignment between modulation of neural excitability and task-specific training.

However, inconsistencies remain in the literature. Some patients with similar injuries achieve overground stepping, whereas others do not [13]. Walking performance can improve without measurable speed changes [9], and functional independence can increase despite minimal physiological changes [26].

We argue that these inconsistencies are due to heterogeneity in residual connectivity rather than methodological error. Current injury classification systems are coarse and do not capture spared synaptic pathways that could support recovery.

Consequently, trial outcomes seem variable because the underlying neural substrates differ. It has been proposed that incorporating physiological and biological biomarkers alongside traditional classifications may improve prognostic precision [63].

Methodological variability further complicates interpretation. Robotic trials tend to use standardized outcome measures, but neural activation is rarely measured, whereas neuromodulation studies offer mechanistic insight but often involve small cohorts using individualized protocols [16].

In some cohorts, HAL^®^ exoskeleton training has been associated with normalization of cortical excitability in the primary somatosensory cortex [64]. Neurophysiological assessments during RAR have confirmed modulation of cortical excitability [65].

Multiple methodological limitations must be considered. These include small cohorts, individualized protocols that limit external validity, robotic trials that often lack mechanistic endpoints, the heterogeneity in the chronicity of the injury, the stimulation parameters, and the training intensity, and finally, publication bias toward positive findings.

In addition, evidence from long-term controlled studies are still limited. Such studies are needed to determine whether improvements extend beyond short-term functional gains and translate into independence, quality of life, and sustained community ambulation.

From a clinical perspective, both approaches have major drawbacks. RAR may not lead to clinically relevant voluntary motor recovery because of insufficient neural drive. on the other hand, neuromodulation often results in highly stimulation parameter-dependent effects requiring external support, and specialized expertise, limiting feasibility for routine clinical practice.

Furthermore, due to the limited follow-up data, it is also unclear whether the neurological improvements during treatment reflect a permanent reorganization or a sustained adaptation requiring continued intervention.

Economic considerations further complicate implementation, as summarized in Table 4. Significant variability exists in cost, accessibility, and infrastructure requirements across interventions.

Taken together, the literature suggests that recovery after SCI depends not only on lesion characteristics but also on the timing, parameters, and engagement of rehabilitation. Theoretical models of recovery should incorporate dynamic interactions between neural excitability and sensory experience rather than treat these domains as independent. Future research should prioritize integrated trial designs, including randomized studies comparing sequential versus simultaneous application of neuromodulation and robotic training. Such approaches may help clarify causal relationships, and move beyond isolated device evaluations of individual technologies.

## 5. Conclusions

The purpose of this review was to merge mechanistic insights with clinical trial evidence to guide rehabilitation approaches in the context of SCI. Existing evidence supports that RAR, through high-dose structured training, improves coordination and functional performance. In contrast, SN may activate residual but functionally silent neural circuits.

Despite the lack of controlled comparative data, early combined paradigms suggest that neural excitability modulation may interact with task-specific training to promote functional recovery. This field is moving from proof-of-concept studies toward more targeted translational frameworks. However, inconsistency in protocols and patient selection currently limit reproducibility.

Further work is needed to stratify physiological responses, standardize stimulation and training parameters and provide longitudinal follow-up to distinguish between neuroplastic and state-dependent adaptations.

### Take-Home Messages for Clinicians

- Recovery after spinal cord injury depends not only on the severity of the lesion but also on the presence of residual neural pathways.
- Robotic rehabilitation improves functional performance but may not restore voluntary control.
- Neuromodulation can enable motor activation, even in severe paralysis, but requires training for functional use.
- Combined approaches (stimulation + training) may offer the most promising results.
- Patient selection and treatment personalization remain the key challenges.
- Long-term functional independence is still not consistently achieved.

## Figures and Tables

**Table 2:** Summary of clinical studies on spinal neuromodulation in patients with spinal cord injury.

Study	Modality	Study Design	Level of Evidence	N	Population	Intervention	Duration	Outcomes	Key Findings
Veith et al. (2025) [43]	EES	Clinical study	Low-Moderate	6	SCI	Spinal stimulation	12 sessions	Functional outcomes, patient perspective	Functional improvements with positive patient-reported outcomes
Angeli et al. (2018) [13]	EES	Case series	Low–Moderate	4	Chronic complete SCI	EES + training	Months	Overground walking	Recovery of walking in motor-complete SCI
Gill et al. (2018) [11]	EES	Case series	Low–Moderate	4	Motor-complete SCI	EES + locomotor training	Months	Independent stepping	Enabled stepping and standing
Gorgey et al. (2023) [14]	EES	Case study	Low	2	Chronic SCI	Percutaneous stimulation	Short-term	Motor control	Rapid activation of motor output
Rejc et al. (2015) [38]	EES	Case series	Low	4	Chronic complete SCI	Lumbosacral stimulation	80 sessions	Standing	Sustained weight-bearing standing
Suggitt et al. (2025) [41]	tSCS	Pilot study (real-world)	Low	10	SCI	Multisite tSCS + activity-based therapy	120 sessions	Safety, function	Safe and effective in community rehabilitation
Wan et al. (2024) [39]	EES	Clinical trial (RESTORES)	Moderate	2	Chronic motor-complete SCI	Epidural stimulation	1 month	Motor control, walking	Recovery of voluntary motor control and overground walking
Rybka et al. (2025) [45]	EES	Pilot	Low	3	Complete SCI	Epidural stimulation	12 months	Motor activation	Feasibility with individualized response
Hankov et al. (2025) [10]	Combined	Pilot	Low	—	SCI	Robotics + neuromodulation	—	Functional recovery	Proof-of-concept for combined approach

Abbreviations: SCI, spinal cord injury; EES, epidural electrical stimulation; tSCS, transcutaneous spinal cord stimulation; N, number of participants.

**Table 3:** Comparison of functional outcomes across robotic-assisted rehabilitation, spinal neuromodulation, and combined approaches.

Domain	RAR	Neuromodulation	Combined Approaches
Walking speed	Limited improvement	Variable response	Moderate improvement
Standing ability	Assisted	Restored in selected patients	Improved
Voluntary movement	Minimal	Enabled	Enhanced
Functional independence (SCIM)	Improved	Variable	Highest
Long-term carryover	Limited	State-dependent	Uncertain

Abbreviations: RAR, robotic-assisted rehabilitation; SCIM, Spinal Cord Independence Measure.

**Table 4:** Cost, accessibility, and limitations of rehabilitation approaches in spinal cord injury.

Intervention	Cost Level	Approximate Cost	Accessibility	Main Limitation
Standard rehabilitation	Low	—	High	Limited neurorestorative effect
Robotic-assisted rehabilitation (RAR)	High	€80–150/session	Moderate	Equipment-dependent
Epidural electrical stimulation (EES)	Very high	>€50,000	Low	Surgical and programming complexity
Transcutaneous spinal cord stimulation (tSCS)	Moderate	—	High	Lower specificity

## Data Availability

No new data were created or analyzed in this study.
